# Unlocking the early fossil record of the arthropod central nervous system

**DOI:** 10.1098/rstb.2015.0038

**Published:** 2015-12-19

**Authors:** Gregory D. Edgecombe, Xiaoya Ma, Nicholas J. Strausfeld

**Affiliations:** 1 Department of Earth Sciences, The Natural History Museum, Cromwell Road, London SW7 5BD, UK; 2 Yunnan Key Laboratory for Palaebiology, Yunnan University, Kunming 650091, People's Republic of China; 3 Department of Neuroscience and Center for Insect Science, University of Arizona, Tucson, AZ 85721, USA

**Keywords:** Cambrian, brains, Chengjiang, Burgess Shale, Arthropoda

## Abstract

Extant panarthropods (euarthropods, onychophorans and tardigrades) are hallmarked by stunning morphological and taxonomic diversity, but their central nervous systems (CNS) are relatively conserved. The timing of divergences of the ground pattern CNS organization of the major panarthropod clades has been poorly constrained because of a scarcity of data from their early fossil record. Although the CNS has been documented in three-dimensional detail in insects from Cenozoic ambers, it is widely assumed that these tissues are too prone to decay to withstand other styles of fossilization or geologically older preservation. However, Cambrian Burgess Shale-type compressions have emerged as sources of fossilized brains and nerve cords. CNS in these Cambrian fossils are preserved as carbon films or as iron oxides/hydroxides after pyrite in association with carbon. Experiments with carcasses compacted in fine-grained sediment depict preservation of neural tissue for a more prolonged temporal window than anticipated by decay experiments in other media. CNS and compound eye characters in exceptionally preserved Cambrian fossils predict divergences of the mandibulate and chelicerate ground patterns by Cambrian Stage 3 (*ca* 518 Ma), a dating that is compatible with molecular estimates for these splits.

## 1. Introduction

Panarthropods provide an intriguing test case for exploring the efficacy of neural characters for inferring phylogeny because the group presents the challenges of unrivalled species diversity and a vast spectrum of phenotypic variation. Euarthropoda alone is known from more than 1.2 million extant species and a fossil record spanning more than 520 Myr. This staggering diversity correlates with marked disparity between major groups, a situation that complicates efforts to resolve the arthropod Tree of Life based on morphological data [1].

However, in the face of this variability, neural architecture within major groups is relatively conserved. As a result, neuroanatomical characters have figured prominently in morphology-based phylogenetic analyses of euarthropod phylogeny [2–4], and the topologies so produced show a high degree of congruence with multi-locus molecular estimates of phylogeny [5–7]. Fossils have likewise been afforded an important role in understanding panarthropod evolution in deep time [8,9]. Especially germane to questions of deep phylogeny are non-biomineralized fossils from the Cambrian that record details not only of exoskeletal form but also appendages and internal organs and tissues. The nervous system has until recently been all but undocumented in the compression fossils that dominate discussion of early panarthropods, named Burgess Shale-type preservation [10–12] after the iconic example of the Burgess Shale in Canada. This dearth of neuroanatomical data from Cambrian fossils has meant that neuroanatomy and palaeontology have largely operated exclusively of each other in considerations of panarthropod evolution. A shortcoming of this for neontological approaches is that the timing of character acquisition of extant groups, e.g. when specific ground pattern characters of the chelicerate and mandibulate nervous systems originated, is unconstrained. Fossils are the only direct basis for elucidating ancient morphologies and dating divergences but until recently the fossil record of neuroanatomy has been largely untapped with respect to phylogenetic questions. Herein we review recent findings in panarthropod ‘neuropalaeontology’ with particular reference to its fossilization and significance for systematics.

Systematic nomenclature herein follows recent recommendations [13]. Panarthropoda encompasses Tardigrada, Onychophora and Euarthropoda; crown-group Euarthropoda is composed of Chelicerata and Mandibulata; that clade and those stem-group euarthropod fossils that possess a structurally differentiated deutocerebral appendage are assigned to Deuteropoda.

## 2. Information loss and retention in the fossil record

The delayed adoption of neuropalaeontology is influenced by a mix of facts and suppositions about decay and its role in information loss in the fossil record. Preservation of internal anatomy in fossils involves stabilization of soft tissue before anatomical detail is lost to decay. Experimental decay series for extant animals are most frequently used to rank tissues into a temporal sequence of their decay proneness, which at one end of the scale are referred to as labile (those lost to decay early) and at the other as recalcitrant (those that withstand considerable decay). The position of particular tissue types in this temporal scale is commonly linked to their fossilization potential [14]. In the extreme case, labile tissues are viewed as unlikely or even impossible to fossilize.

Decay experiments have been conducted on many kinds of invertebrates, the most phylogenetically relevant for this study being those on marine decapod crustaceans [15], branchiopods [16] and on onychophorans [17]. The latter classified the nerve cords as relatively labile and this observation underpinned the authors' scepticism about the preservation potential for neural tissue in panarthropod fossils in general and in Early Palaeozoic Konservat-Lagerstätten in particular.

Some caveats need be acknowledged. The same decay experiments demonstrate that muscle is a labile tissue and so by the same standard we could be led to predict its low fossilization potential. However, diverse taxa from Mesozoic and Cenozoic lithographic limestones, including arthropods, molluscs and fishes, show that muscle can be preserved in fine detail by being replicated by calcium phosphate [18]. Such apatite replication of muscle is represented over a considerable span of the geological record and is not confined to ‘Solnhofen-type’ preservation in platy limestones. Early diagenetic replication of muscle in calcium phosphate is observed in Cambrian Konservat-Lagerstätten, including Sirius Passet in Greenland [19], the Emu Bay Shale in Australia [20] and the Burgess Shale in Canada [21]. Likewise, the onychophoran decay experiments show that the gut is prone to early decay [17], yet the fossil record of the Cambrian is replete with guts [22–24]. In Burgess Shale-type fossils, decay-resistant structures such as the cuticle and the appendages are preserved effectively as two-dimensional carbonaceous compressions; conversely and almost paradoxically, a more labile tissue type, the gut, is frequently preserved in three dimensions [19]. The volatility of the gut serves to localize microbial activity that leads to early diagenetic mineral precipitation [15], morphology again typically being replicated in calcium phosphate [10]. The fidelity of preservation is such that sub-millimetric structures can be resolved in, e.g. the midgut glands of Cambrian arthropods [23,24]. These observations on muscle and the gut of course have no necessary bearing on the fossilization potential of neural tissue (exceptional preservation of muscle and gut could, for example, be dependent on a single taphonomic mode, i.e. phosphatization). They do nonetheless reveal that decay proneness does not in itself equate with non-fossilization. Perhaps, the most stunning example that belies the notion that neural tissue rapidly decays is the discovery of a human brain preserved almost intact over more than 2000 years in the absence of entombment [25].

To explore aspects as to how decay and compaction affect neural tissue preservation, actualistic experiments were conducted on extant proxies, as described in §2*a,b*, and compared with putative fossilized CNS.

### (a) The effect of sediment on neural tissue preservation

Recently, the consequences of differences in sediment mineralogy on the preservation of non-biomineralized recalcitrant tissues in annelids and arthropods have been explored experimentally [26]. In a similar vein, we have conducted experiments with the polychaete *Nereis virens* to examine the survival of neural tissue in carcasses that have been entombed in sediment and subjected to compaction during the decay process (cf. observations on decay of *N. virens* in artificial seawater) [27].

**Figure 1.**
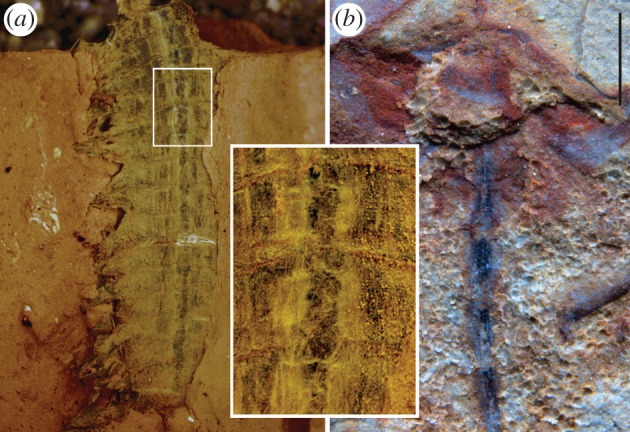
Entombment experiment with *Nereis virens* compared with fossilized CNS in the Cambrian euarthropod *Chengjiangocaris*. (*a*) *N. virens* after compression and desiccation in clay. Inset shows ventral nerve cord. (*b*) *Chengjiangocaris kunmingensis* [28], showing ventral nerve cord with segmental ganglia in the trunk (image courtesy of J. Yang, J. Ortega-Hernández and X. Zhang). Scale bar, 1 mm.

In our entombment experiments, living specimens of *Nereis virens* were kept in seawater at 7°C. Animals were immobilized on ice. Heads with about 1–1.5 cm of the trunk were placed lengthwise on the surface of a 1.0 cm deep layer of fine-grained Carbondale C clay powder soaked in seawater. This was contained in a container approximately 9 cm long, 3.5 cm wide, and 5 cm deep, lined with Teflon film. The polychaete was then covered with a slurry of clay in seawater to a depth of 2.5 cm. The surface was covered with Teflon on which was placed an 8 × 3 cm glass slide. The container and its contents were transferred to the cool room (7°C) and a 2 × 6 × 2 cm brass weight (80 g) was placed on the slide. The container was left in this condition for 14 days, after which the weight was increased to 160 g, and then to 240 g after a further 14 days. This ensemble remained in this condition for one to two months, during which the clay slowly dried. The weights were then removed and the container transferred to room temperature and left for a further 30–40 days. The slide and Teflon were then removed and the hardened clay removed from the container. The clay was cracked open with a chisel until flattened dewatered remains were found as part and counterpart, one of which came away as almost exclusively cuticle; the other contained traces of soft tissue (muscle and ganglia; figure 1*a*). Excess grains of clay were removed with a fine water-colouring brush prior to photography.

These experiments reveal that the nerve cord clearly retains its integrity months after death, with discrete segmental ganglia being obvious (figure 1*a*). Such simulations do not of course reproduce the process of fossilization. Nonetheless, the durability of neural tissue in sediment suggests that decay experiments designed to exclude sediment from the equation are probably ignoring a vital factor in exceptional fossil preservation.

### (b) The effects of flattening on brain shape

When interpreting the anteroposterior sequence of neuromeres and segmental tracts in the brains of compression fossils such as the Cambrian examples outlined in §3, there is a need to consider the three-dimensionality of arthropod brains and the potential for topologies to be distorted by compaction.

**Figure 2.**
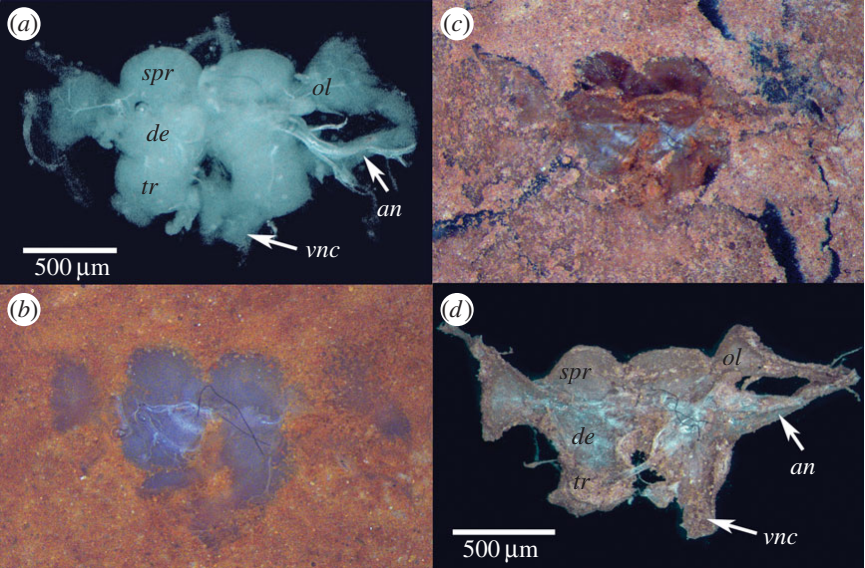
Experimental flattening of cockroach brain. (*a*) Fresh excised brain of *Periplaneta americana*. (*b*) Brain embedded in slurry of wet clay. (*c*) Compacted, drying brain in clay. (*d*) Flattened brain removed from clay. *spr*, superior protocerebrum; *de*, deutocerebrum; *tri*, tritocerebrum; *ol*, optic lobe; *an*, antennal nerve; *vnc*, ventral nerve connective.

To perform an actualistic experiment, fresh excised brains of the cockroach *Periplaneta americana* (figure 2*a*) were placed with antennal nerves downward on a slurry of Carbondale C clay contained in the well of a thick glass slide. Wet clay was then placed over the brain and a heavy glass cover placed on top (figure 2*b*). The ensemble was left in the cold room for 10 days during which the clay slowly dried. Afterwards, the glass cover was removed and the dried clay carefully brushed away until dewatered tissue was encountered as an extremely thin film of weakly stained material (pinkish blue) not thicker than lens tissue (figure 2*d*). Control experiments, in which whole heads were embedded, revealed similarly preserved and likewise paper-thin brains.

In both experimental conditions, brain profiles did not differ from those of fresh material at the same orientation (figure 2). This suggests that flattening is not likely to lead to erroneous interpretations of the serial register of brain segmentation.

## 3. The fossil record of panarthropod neural tissue

It is demonstrably incorrect that neural tissue cannot fossilize. The brain is preserved in various kinds of insects from Miocene amber of the Dominican Republic [29,30], including species of Hymenoptera, Diptera and Coleoptera. Although the histological details of neural tissue in these amber fossils are uncertain, brain outlines preserve accurately. Even older ambers, notably the Eocene Baltic amber, likewise preserve the outlines of neural tissue in sufficient fidelity for imaging approaches such as synchrotron microtomography to resolve the brain, optic neuropils, antennal nerves and suboesophageal ganglion in the case of a strepsipteran [31]. Acknowledging that amber is geologically young and possibly the ‘gold standard’ of fossilization (e.g. preserving the mitochondria of flight muscle in insects that also preserve the brain) [30], it is nonetheless special pleading to discard amber as a whole in order to defend the case that neural tissue has no fossil record.

In recent years, the search for fossilized neural tissue has shifted much deeper in geological time and to a different mode of fossil preservation, specifically to Cambrian fossils of Burgess Shale-type preservation. They are of particular evolutionary importance because they sample animal lineages early in their phylogenetic history.

The Burgess Shale in British Columbia, Canada [32], dates to Cambrian Stage 5, spanning an interval from *ca* 510–505 Ma. Similar taxa and styles of preservation occur in the broadly coeval units throughout the region, such as the recently documented Marble Canyon assemblage [33]. Neural tissue preservation in the Burgess Shale has until recently been rather sporadic in its documentation, though its history of study extends to the 1970s. Early reports include two specimens of the common priapulid *Ottoia prolifica* in which a paired reflective strand along the ventral midline was interpreted as the nerve cord [28] (figure 2*a*), its paired nature possibly representing the margins of a single cord. It is most plausibly situated ventrally, as expected for priapulids. The problematic organism *Amiskwia sagittiformis* Walcott has likewise had reflective traces in the head and trunk identified as possible cerebral ganglia and the nerve cord, respectively [34].

The other major occurrence of neural tissue preservation in Cambrian fossils is the Chengjiang biota of Yunnan Province, China [35]. This early Cambrian Konservat-Lagerstätten predates the Burgess Shale by some 10 Myr, dating to Cambrian Stage 3. CNS has also been documented in the Xiaoshiba Konservat-Lagerstätten, in the Hongjingshao Formation in Yunnan Province [36]. This biota slightly postdates Chengjiang in Cambrian Stage 3.

**Figure 3.**
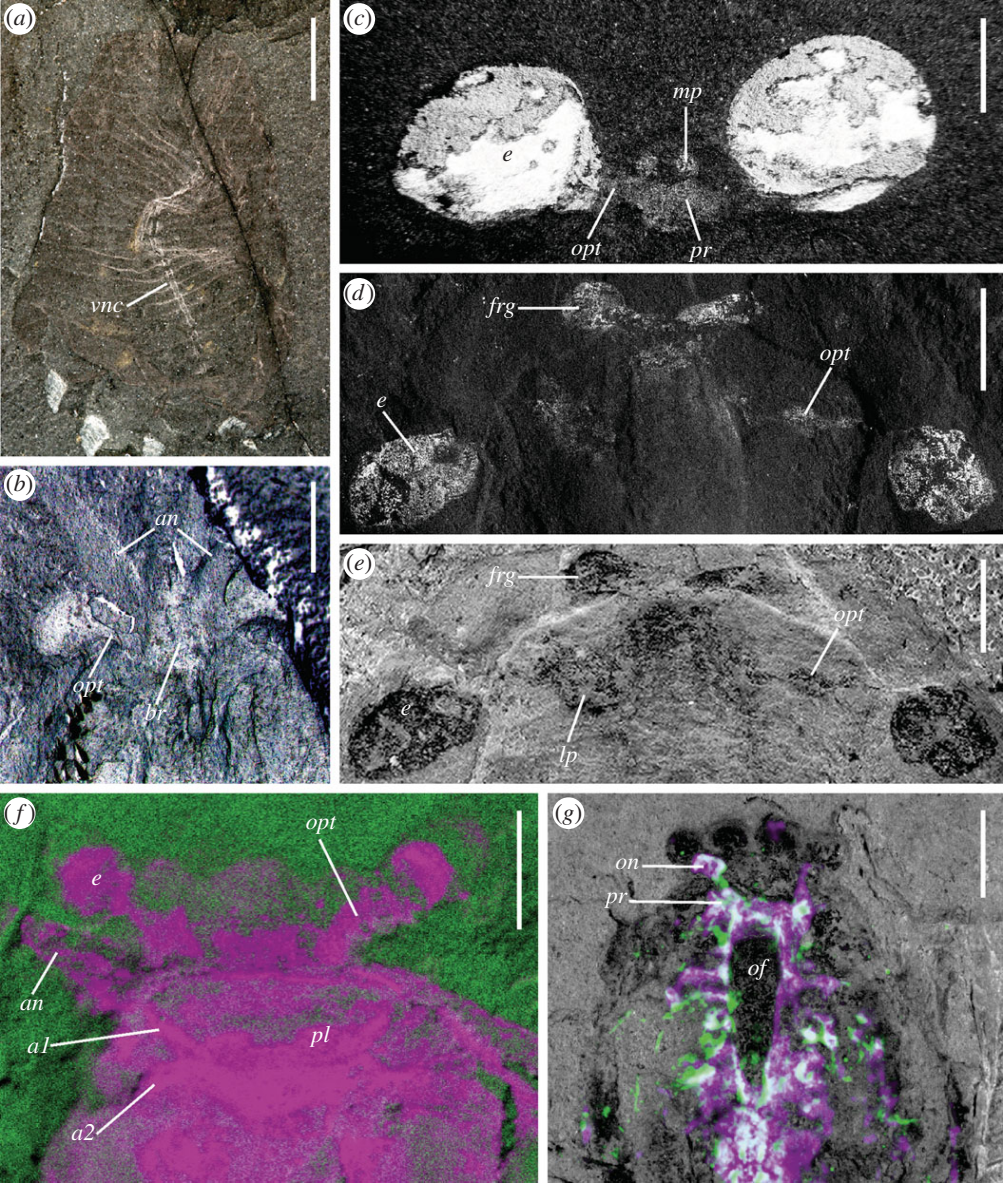
Cambrian Burgess Shale-type fossils preserving traces of the CNS. (*a*) *Ottoia prolifica*, USNM 188635, showing ventral nerve cord (*vnc*) as paired strands. (*b*) *Waptia fieldensis*, USNM 83948j, anterior part of head with inverted light. (*c*) *Odaraia expansa*, ROM 60746, anterior cephalic structures in cross-polarized light (image from http://burgess-shale.rom.on.ca/en/index.php, courtesy of Jean-Bernard Caron, Royal Ontario Museum). (*d,e*) *Lyrarapax unguispinus*, YKLP 13305, anterior part of head: (*d*) SEM-EDX carbon map; (*e*) light photograph. (*f*) *Fuxianhuia protensa*, YKLP 15006a, anterior cephalic structures, micro X-ray fluorescence iron map (lavender). (*g*) *Alalcomenaeus* sp., YKLP 11075, head, neural traces in inverted white coincidence signal of micro-CT (green) and SEM-EDX iron map (magenta). *a1*, antennal (deutocerebral) tract; *a2*, tritocerebral tract; *an*, antenna; *br*, brain; *e*, eye; *frg*, frontal ganglion; *lp*, lateral protocerebrum; *mp*, median photoreceptor; *of*, oesophaegeal foramen; *on*, optic neuropil; *opt*, optic tract; *pl*, protocerebral lobe; *pr*, protocerebrum. Scale bars, 2 mm except *a*, 5 mm.

The §3*a–i* summarizes work to date on panarthropod CNS in the Burgess Shale and Chengjiang/Xiaoshiba (figure 3). Representative species are listed following a stemward to crownward sequence with reference to Euarthropoda (following the nomenclature and tree topology of Ortega-Hernández [13]).

### (a) Paucipodia inermis

**Figure 4.**
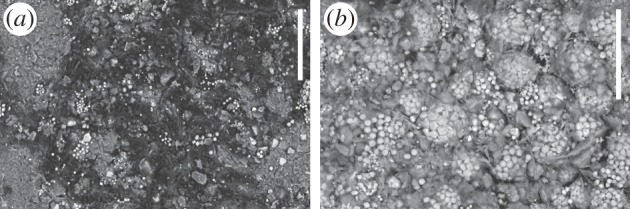
Neural tissue preservation in *Fuxianhuia protensa*. (*a*) Carbon on nerve track from deutocerebral region (YKLP 15006b, counterpart); (*b*) pyrite framboids and crystals from eye region (YKLP 15006a, part). Scale bars, 20 µm.

This lobopodian from Chengjiang includes specimens with paired longitudinal bands spanning several trunk segments. The bands are associated with bilaterally symmetrical rounded swellings preserved with similar violet pigmentation ([37], fig. 4*f*). These structures were interpreted as the nerve cord and segmental ganglia, respectively [37]. One specimen has paired pigmented lobes in the preoral region ([37], figs 3*a* and 4*g*) that were tentatively identified as the brain. However, differences in the style of preservation from CNS in other Chengjiang taxa described in §3*b*–*d,g* have prompted questions about the identity of the putative neural tissues in *Paucipodia inermis* [38].

The most recent phylogenetic analysis of Cambrian lobopodians in the context of panarthropod phylogeny resolves *P. inermis* (as well as most of the ‘armoured’ lobopodians) as stem-group Onychophora [38]. If the CNS interpretations [37] and the phylogenetic placement are accurate, segmental ganglia in a stem-group onychophoran present a closer similarity to the segmentally ganglionated nerve cords of euarthropods and tardigrades than to extant Onychophora. The onychophoran nerve cord has been viewed as non-ganglionated [39], and the brain is debatably composed of fused ganglia [40].

### (b) Lyrarapax unguispinus

Radiodonta, best known from *Anomalocaris* [21], are large Cambro-Devonian predators that have been placed in various parts of the ecdysozoan tree, ranging from having closest affinities to Cycloneuralia (with convergent similarities to arthropods), being a part of the euarthropod stem group, or belonging to the euarthropod crown group as stem-group chelicerates (reviewed by [8]). Controversy has surrounded the segmental affiliation of their anteriormost appendages and whether they are or are not aligned with those of other fossil groups.

Species of various radiodontans from the Burgess Shale and Chengjiang indicate that these animals have a pair of stalked eyes that project from the dorsolateral part of the head [21,41]. In these biotas, the inferred eyes are preserved as carbon films that lack details of the visual surface. Specimens from Australia clarified the visual surface of *Anomalocaris* [42]. Lenses are very numerous, more than 16 000 ommatidia per eye, and they have a hexagonal packing arrangement that corresponds closely to various kinds of apposition eyes seen in extant euarthropods. Ommatidial lenses are also known in the Devonian *Schinderhannes* [43], which has been identified as a radiodontan [13,44]. The discovery that radiodontans have compound eyes strengthens the case for euarthropod affinities and constrains the node at which such organs evolved in the euarthropod stem group.

*Lyrarapax unguispinus*, a radiodontan from Chengjiang with an enlarged, paddle-like anterior trunk flap, also preserves the eyes, and the carbon signal associated with them shows relationships that indicate the preservation of neural tissue [45]. A transverse tract extending from the eye represents the optic nerve (figure 3*d,e*). Assuming the same segmental relationships as eyes have in all panarthropods, this constrains the interpretation of the protocerebral part of the brain. A pair of carbon spots preserved similarly to the retinae is located in the anteromedian part of the head (figure 3*d*). Based on a common mode of preservation and their connection to the protocerebrum, these paired structures are taken to be parts of the nervous system. Their pre-ocular position corresponds to the origination of a pre-ocular appendage pair, the robust, spinose frontal appendages. The reflective patches are thus interpreted as a pair of frontal ganglia associated with these appendages [45].

Extant euarthropods lack frontal appendages and frontal ganglia, but it has been suggested that onychophorans possess a segmentally homologous appendage pair, the so-called antennae or frontal appendages [46], and neuropil areas associated with these appendages have been identified as ganglia [47].

### (c) Fuxianhuia protensa

*Fuxianhuia protensa* is a member of a clade from the early Cambrian of South China collectively known as Fuxianhuiida. The group has been resolved in the euarthropod stem group in numerous phylogenetic analyses [44,48,49]. Bergström *et al.* [49] documented two specimens of *F. protensa* from the Chengjiang biota that they suggested preserve possible traces of CNS. One of these was drawn upon to interpret brain morphology in this species [50]. The structure in question is situated medially in the head (figure 3*f*). Its anteromedial outline is a pair of symmetrical lobes, interpreted as protocerebral. It throws symmetrical tracts into paired appendages, specifically the antennae and a specialized post-antennal appendage pair [36]. The same style of preservation is seen in tracts that extend through the optic lobes, interpreted as the optic nerve. In comparison to small brains in extant crustaceans such as shrimps, *F. protensa* is interpreted as having proto-, deuto- and tritocerebral neuromeres in its pre-stomodaeal brain.

Putative neural tissue in the original *F. protensa* specimen shows pronounced enrichment in iron [50] (figure 3*f*). Scanning electron microscopy revealed that the iron in the brain and optic lobes is in the form of framboids of pyrite crystals (figure 4*b*). As in Chengjiang fossils generally, iron oxide and iron hydroxide pseudomorphs follow pyrite [51,52], sulfur having been depleted in the weathering process. The framboidal, crystalline morphology has been interpreted as early diagenetic pyrite [51], suggestive of neural tissue having been replicated by pyrite mineralization. The counterpart, however, shows carbon between the pyrite framboids (figure 4*a*), as would be consistent with an original carbon template and subsequent tissue-specific localization of pyrite. A role for carbon in preservation of neural tissue in *F. protensa* is also seen in other specimens, in which energy dispersive X-ray spectroscopy (SEM-EDX) depicts a strong carbon signal in the inferred brain and nerve tracts. In these cases, iron is instead preferentially concentrated in recalcitrant extracellular tissues like cuticle, as is commonly the case in Chengjiang fossils [51,52]. It has been proposed that relocalization of iron in Chengjiang fossils occurred late in diagenesis [53], but the diagnostic pseudomorphs in the brain of *F. protensa* argue against pyrite not having played a role in fixation of these tissues. The interplay between carbon and pyrite in the preservation of the same kind of soft tissue has come to be recognized as a pervasive pattern in Ediacaran–Cambrian Konservat-Lagerstätten, the dynamic between the two taphonomic modes sensitive to variation in bacteria over minute spatial and temporal scales [54].

### (d) Chengjiangocaris kunmingensis

In addition to the brain of *F. protensa*, parts of the nervous system have been documented in another fuxianhuiid taxon, *Chengjiangocaris kunmingensis*, from the early Cambrian Xiaoshiba site. This material [36] shows the ventral nerve cord as the expected arthropod rope-ladder nerve cord with a pair of ganglia in each segment (figure 1*b*).

### (e) Odaraia alata

The bivalved Burgess Shale arthropod *Odaraia alata* is currently considered to be a member of the euarthropod stem group, closely allied to a number of other bivalved Cambrian taxa that are resolved as a paraphyletic assemblage stemward of fuxianhuiids [44]. Neuroanatomy has been documented in the head of *O. alata* [55], with traces of the brain represented in multiple specimens. A pair of large lateral eyes is preserved as highly reflective carbon films, and reflective tracts extending from the eyes (the optic nerves) converge medially on a bilateral symmetrical bilobate field that has the same style of preservation (figure 3*c*). The confluence with the eyes, bilateral symmetry and correspondence in position and relationships to a protocerebrum underpin the interpretation of the medial reflective area as the brain [55]. Three reflective spots are bilaterally symmetrically positioned anterior to the inferred protocerebrum and are connected to it. They are evidently associated with a small triangular sclerite (‘anterior sclerite’ *sensu* [56]) that lies between the anteromedial notch between the carapace valves. The trio of spots has been interpreted as medially positioned photoreceptors [55].

### (f) Mollisonia symmetrica

Optic neuropils have been reported in *Mollisonia symmetrica*, a Burgess Shale arthropod of undetermined affinities from the Marble Canyon locality ([33], figure 3*p*,*q*).

### (g) *Alalcomenaeus* sp.

*Alalcomenaeus* and the well-known *Leanchoilia* [57] belong to a controversial group of Cambrian arthropods known as Megacheira, or great-appendage arthropods. The colloquial name is derived from their anteriormost appendage pair, which has three spine-like podomeres that in the relevant species each bear a flagellum [57]. Partly depending on the segment with which the great appendage is affiliated, megacheirans are variably identified as stem-group euarthropods [44] or as crown-group euarthropods related to chelicerates. The latter scheme homologizes the structure of a chelicera and the great appendage. This is based on shared possession of an elbow joint that separates a basal pedunculate part and a distal part with extensions that contribute to a chela composed of a fixed and a movable finger [58,59].

Neuroanatomical interpretations are based on a specimen of the Chengjiang megacheiran *Alalcomenaeus* sp. [60]. It is preserved in dorsal view, with internal and external structures both subjected to pyritization. Overlaying the iron signal from the pyrite with the density difference detected by micro-CT permits a profile identifiable as trace nervous system to be visualized (figure 3*g*). It depicts continuity between the eyes (paired on each side of the head) and a swelling that corresponds to the position and morphology of an optic neuropil. The latter is connected to a region bordering an elongate oesophageal foramen that has the expected relationships of the protocerebrum of chelicerates. This is then followed caudally by more diffuse neural tissue that includes ganglia associated with the segmental appendages of the head and trunk.

A single optic neuropil separated from the protocerebrum and serving paired eyes is shared with Chelicerata among living arthropods. A concentration of neural tissue in the post-ocular region is associated with the attachment site of the great appendage. This alignment of head segments is consistent with a homology of chelicerae and great appendages as deutocerebral appendages.

### (h) Helmetia expansa

*Helmetia expansa* from the Burgess Shale belongs to Conciliterga, a Cambrian clade within a group of Palaeozoic taxa that includes trilobites. Depending on its exact composition, the names Lamellipedia [61] or Artiopoda [62] are applied to this ‘trilobitomorph’ assemblage, and their affinities to Mandibulata or Chelicerata remain contested.

As described above for *O. alata*, *H. expansa* has an anterior sclerite positioned anteromedial to the cephalic shield. This sclerite is associated with a pair of carbonaceous reflective spots or ‘frontal organs’ that are, as for *O. alata*, interpreted as photoreceptors [55]. Also as for *O. alata*, the eyes share this same style of preservation as highly reflective organs and the optic nerve is traceable medial to the eye. The common pattern of the anterior sclerite in *Odaraia* and *Helmetia* being associated with frontal organs that are, at least in the case of *O. alata*, demonstrably connected to the protocerebrum, underpins a homology of the sclerite itself between these stem- and crown-group euarthropods [55].

### (i) Waptia fieldensis

Neural tissue preservation has been identified in a crustacean-like arthropod from the Burgess Shale, *Waptia fieldensis* [63,64]. The species has not been comprehensively revised in modern times and whether its similarities to crustaceans are indicative of close affinities in the euarthropod crown group is unresolved. Nonetheless, the fossils preserve mechanosensory and chemosensory sensilla on their appendages that can be interpreted both structurally and functionally in comparison to malacostracan crustaceans. In some specimens, the optic nerve can be traced through the eye stalk [63] to a comparably pigmented median region that corresponds to the position and expected morphology of a brain [64] (figure 3*b*).

## 4. Conclusion

The interpretations of Cambrian fossils summarized herein set constraints on the timing of nervous system evolution in euarthropods. If, for example, *Alalcomenaeus* and other Megacheira are total-group chelicerates, then the chelicerate and by extension mandibulate ground patterns are at least as old as it, or some 518 Myr. The standard placement of fuxianhuiids in the euarthropod stem group [44,48,49] suggests that mandibulate-like characters of *F. protensa*, such as three neuromeres in the pre-stomodaeal brain and nested optic neuropils [50], may resolve more deeply in the euarthropod total-group than conventionally assumed. Placing *L. unguispinus* and fellow radiodontans more basally in the euarthropod stem, pre-ocular frontal appendages and frontal ganglia emerge as characters that were probably present in the deepest parts of the euarthropod stem lineage.

Molecular dating interprets the deep nodes of crown-group lineages of Euarthropoda as having evolved by the Cambrian [7,65,66]. This draws on calibrations that identify particular early Cambrian fossils as crown-group euarthropods, such as the crown-group pancrustaceans *Yicaris* [67] and *Wujicaris* [68] from Cambrian Stage 3, and total-group branchiopods known from small carbonaceous fossils [69] as early as Cambrian Stage 4. The findings from neuropalaeontology are likewise consistent with clades such as total-group Chelicerata and Mandibulata being present in the main burst of the Cambrian explosion and having already evolved diagnostic characters of brain organization.
